# Evolution of plant metabolism: the state-of-the-art

**DOI:** 10.1098/rstb.2023.0347

**Published:** 2024-09-30

**Authors:** Alisdair R. Fernie, Sophie de Vries, Jan de Vries

**Affiliations:** ^1^ Max-Planck-Institute of Molecular Plant Physiology, Am Mühlenberg 1, Potsdam-Golm 14476, Germany; ^2^ Department of Applied Bioinformatics, University of Goettingen, Institute of Microbiology and Genetics, Goldschmidtstr. 1, Goettingen 37077, Germany; ^3^ University of Goettingen, Campus Institute Data Science (CIDAS), Goldschmidstr. 1, Goettingen 37077, Germany; ^4^ Department of Applied Bioinformatics, University of Goettingen, Goettingen Center for Molecular Biosciences (GZMB), Goldschmidtstr. 1, Goettingen 37077, Germany

**Keywords:** evolution, primary metabolism, phytohormone, secondary metabolism

## Abstract

Immense chemical diversity is one of the hallmark features of plants. This chemo-diversity is mainly underpinned by a highly complex and biodiverse biochemical machinery. Plant metabolic enzymes originated and were inherited from their eukaryotic and prokaryotic ancestors and further diversified by the unprecedentedly high rates of gene duplication and functionalization experienced in land plants. Unlike prokaryotic microbes, which display frequent horizontal gene transfer events and multiple inputs of energy and organic carbon, land plants predominantly rely on organic carbon generated from CO_2_ and have experienced relatively few gene transfers during their recent evolutionary history. As such, plant metabolic networks have evolved in a stepwise manner using existing networks as a starting point and under various evolutionary constraints. That said, until recently, the evolution of only a handful of metabolic traits had been extensively investigated and as such, the evolution of metabolism has received a fraction of the attention of, the evolution of development, for example. Advances in metabolomics and next-generation sequencing have, however, recently led to a deeper understanding of how a wide range of plant primary and specialized (secondary) metabolic pathways have evolved both as a consequence of natural selection and of domestication and crop improvement processes.

This article is part of the theme issue ‘The evolution of plant metabolism’.

## 1. Introduction

**Figure 1.**
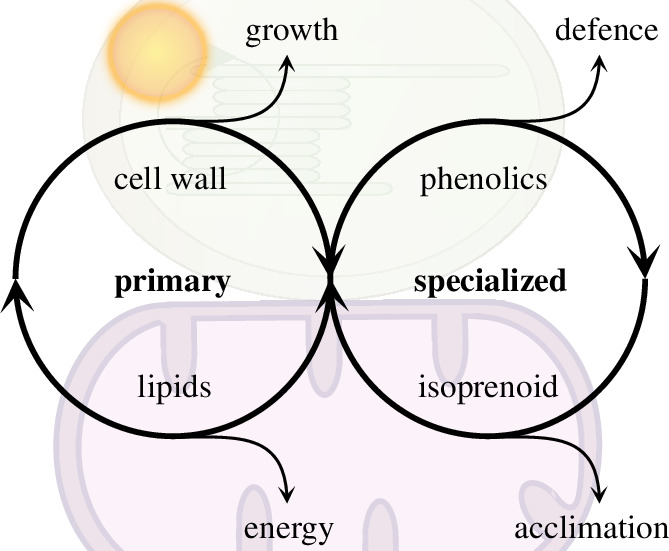
A schematic diagram displaying the evolution of primary and secondary plant metabolites for energy, growth, defence and acclimation.

A long-standing goal of biology has been to understand how the complex biomolecular networks underpinning life attained the forms that we observe today [1,2], with cellular metabolism arguably being one of the earliest such networks [3]. However, our study of the evolution of metabolism and physiology (evo-physio) [4] greatly lags behind that of the evolution of development (evo-devo). That said, given that metabolism underlies developmental processes [5], it is in fact a part of evo-devo [1]. Moreover, metabolism is central in response to many other cues including the adaptation to abiotic and biotic factors and as such it is subjected to natural selection and/or shaped by crop domestication [6–9]. Indeed, within the metabolic network, many thousands of biochemical processes are linked in tailored systems, which have been subject to the action of billions of years of evolution [1]. Here, we will limit ourselves to the evolution of plant primary metabolism alongside the emergence of plant-specific specialized metabolism and their subsequent diversification. We refer the interested reader to a range of research articles and reviews regarding the origin of metabolism itself [10–18] and rather chose the mosaic origin of plant primary metabolism as the start point of this article. Following this, we review recent evolutionary diversification of plant primary metabolism. While covering the central energy pathways of plants in brief below, we here focus on pathways beyond these as we feel that they have been much discussed elsewhere; instead, we discuss the pathways of sugar, lipid and amino acid metabolism instead. Having covered central primary metabolism, we next detail advances in understanding of hormone biosynthesis and perception and signal transduction. Finally, we discuss the evolution and diversification of specialized metabolism. Here, we attempt to provide a broader coverage selecting to provide updates into phenylpropanoid, alkaloid and terpene metabolism. In discussing these different classes of metabolites, we will briefly outline the similarities and differences in their evolution and consider how the abiotic and biotic environment factors into their evolutionary history (figure 1). However, we will return to this in more detail in the concluding article of this special issue [19]. In addition to the above-described pathway-centric chapters, we will also highlight a handful of methodological developments that have in the past decade or so greatly facilitated advances in a broad range of species, paying particular attention to next-generation sequencing data [20], combining phylogenomics and synteny [21], metabolomics [22,23] and the approach of ancestral protein reconstruction [24–26]. At least the first three of these are now frequently used in the study of plant primary, hormonal and specialized metabolism and as we detail below, ancestral protein reconstruction has been used in the study of both hormonal and specialized metabolism—as well as core functions of photosynthetic organisms [26]. In the next section, we give a short introduction on the evolution of the green lineage and then briefly outline of the scope the articles that we invited for this collection, as well as documenting our initial intentions on assembling it.

### (a) Streptophyte evolution and the origin of the terrestrial macroflora

**Figure 2.**
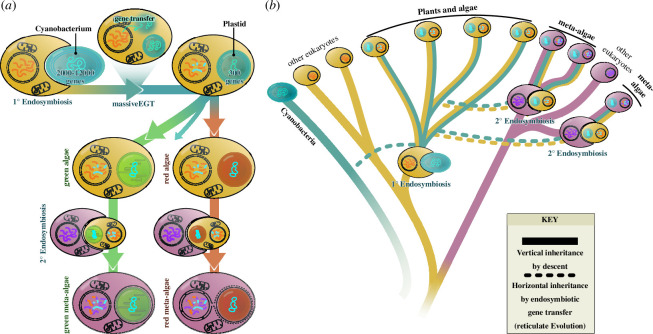
Endosymbiosis and the evolution of plants and algae. A cladogram of the diverse photosynthetic lineages. 1° = primary; 2° = secondary or higher order.

All plants and algae ultimately trace their origin to a pivotal endosymbiotic event. Here, a free-living cyanobacterium was engulfed by a heterotrophic protist, giving rise to the lineage of Archaeplastida, whose last common ancestor likely had most features of a typical extant alga [27–29]. Additional, secondary (or higher-order) endosymbiotic events that involved different eukaryote hosts and an archaeplastidal alga as the endosymbiont gave rise to the lineages of algae (sometimes called meta-algae) with complex plastids surrounded by more than two membranes, such as diatoms (2° red), euglenids (2° green) and many more [30–33]. The Archaeplastida diverged into three major lineages: the Glaucophyta, Rhodophyta (red algae) and Chloroplastida (the green lineage) (figure 2). Most articles in this special issue focus on members of the Chloroplastida, which diversified into Prasinodermatophyta, Chlorophyta and Streptophyta.

The lineage of Streptophyta is unique in that it harbours the only group of photosynthetic organisms that was able to conquer the terrestrial habitat on a global scale and rise above its substrate: the land plants (Embryophyta) [4,34–38]. This event happened in a timeframe of 510–600 Ma [39,40], during which the streptophyte algal progenitors of land plants colonized the bleak terrestrial habitat. Collectively, these steps are referred to as plant terrestrialization. Given its global impact, there is a series of fascinating questions that revolve around the plant terrestrialization event. The recent recovery of a deep split of land plants, resulting in the two monophyletic clades Bryophytes and Tracheophytes, significantly changed our power to predict the characteristics of the last common ancestor of land plants—and with that, our picture of the evolutionary trajectory of any given trait [41,42]. The different phylogenomic studies robustly recovering Zygnematophyceae as the sister group to land plants [41,43–46] are allowing us now to trace those characteristics that the conquerors of land already possessed. Overall, this has established a robust framework for studying the deep evolutionary roots of land plants and started major endeavours into the biology of land plants’ algal relatives [47–52].

In order for embryophytes to conquer the land, the earliest land plants must have been able to combat the various adverse challenges posed by their new habitat from their first step on land [4]. Here, the early land plants likely benefitted from a range of traits. For one, they very likely had the ability to engage in mutualistic symbiosis with fungi [53,54] for obtaining vital nutrients, and for another, they must have been able to adequately respond to environmental stressors. Many of these are still major challenges faced by extant plants today, and considered as contributors to the evolution of plant metabolism diversity [55].

### (b) The mosaic origin of primary plant metabolism and its subsequent evolution

The genomes of Archaeplastida are derived from at least three origins. The ancestral archaeal host, which acquired mitochondria via endosymbiosis of an α-proteobacterial progenitor that was either *Rickettsia*-related [56] or sister to all α-proteobacteria [57,58], followed by a further endosymbiosis of a *Gloeomargarita*-related or section V cyanobacterial ancestor that gave rise to the plastid [59–62]. Since all three lineages had the majority of enzymes involved in primary metabolic pathways, the pathways retained in land plants are the result of a pick-and-mix of genes and enzymes of different origin. This had several consequences: first, given that the two endosymbiotic events resulted in multiple subcellular compartments, a remarkable expansion including multiple redundant pathways of core metabolism occurred. This then resulted in the majority of plant primary metabolism being of a highly mosaic ancient origin [63,64]. This is well-known for pathways including the respiratory pathways of glycolysis [65], the oxidative pentose phosphate pathway [66] and the tricarboxylic acid (TCA) and Calvin–Benson–Bassham cycles [1]. Similarly, galactolipids are ubiquitous in the photosynthetic membranes of cyanobacteria, algae and land plants, being synthesized in plants via both endoplasmic reticulum and plastid pathways, although the relative contributions thereof are species-dependent [67,68], while the evolution of plastidial and extra-plastidial pathways of isoprenoid biosynthesis has additionally been subject to quite some research [69,70]. Given that the evolution of these pathways has previously been extensively reviewed, we will not detail them here but rather outline basic knowledge concerning sugar, amino acid and lipid metabolism, which are the subject of individual articles in this collection. Sugar metabolism, while key to plant energy metabolism, is phylogenetically more diverse than the other key respiratory pathways with differences in the way plants store carbon, transport it and break it down—the majority of which are the consequence of evolutionary events following endosymbiosis. That said, sucrose biosynthesis—which is common to both plants and the ancestral organelle progenitors—clearly originated in the proteobacteria or a common ancestor of proteo- and cyanobacteria [71], with recent studies on the invertase gene family suggesting that the organellar invertases were of a similar origin as well as potentially being important for the thriving of land plants [72]. Recent studies have indicated that a key role in sugar signalling is performed by trehalose and its phosphorylated metabolite trehalose 6-phosphate. These metabolites were for a long time believed to be absent in plants—yet far from being absent they appear to have taken on a key role in signalling, leading to suggestions that at least trehalose 6-phosphate could even be regarded as a phytohormone [73]. Plant amino acid biosynthesis pathways tend to be localized to the plastid but many of them are derived from non-cyanobacterial origins. While potential phylogenetic artifacts must be carefully evaluated [74], plants and algae likely acquired some of the enzymes by endosymbiotic gene transfer and the others via horizontal gene transfer (HGT), with enzymes of aromatic amino acid biosynthesis being derived from at least six different sources; frequent HGT in prokaryotes, however, complicates pinpointing the donors of such acquisitions, since the endosymbiont also had chimeric genome riddled with ancient HGTs and HGTs that have been (and still are) consistently reshaping bacterial genomes for billions of years [75]. Regardless, this means that the enzymes required for the biosynthesis of plant amino acid biosynthesis or, for example, of phylloquinone and tocopherols [76,77] were not simply acquired from cyanobacterial counterparts.

Primary metabolism is largely assumed to be conserved, especially within the plant kingdom which relies on photosynthetic carbon fixation as the primary source of organic carbon and energy and additionally has similarly limited primary sources of nitrogen. This notion is also true in comparison with the highly diversified specialized metabolism of the plant kingdom, which has been estimated to comprise upwards of a million metabolites [78]. Despite this fact, there are some—albeit relatively rare—alterations in lineage-specific primary metabolism, which likely have considerable impact on the overall metabolic networks and physiology of specific plants. Particular examples here include photosynthetic modes, the relative subcellular operation of the TCA cycle, sugar transport and catabolic pathways and lineage-specific amino acid biosynthetic pathways. The evolution of C_3_, C_4_ and Crassulacean Acid Metabolism (CAM) modes of photosynthesis have been well covered at both anatomical [79,80] and biochemical/molecular levels [81–84], so we will not detail them here, yet would like to highlight the recently reported alternative photosynthetic pathways that drive the algal CO_2_ concentrating mechanism [85]. Additionally, in some species—namely those in the family of the Brassicaceae, which includes Arabidopsis—a cytosolic fumarase is highly active [86], and fumarate represents a major form of carbon transport [87]. Similarly, certain species from the Cucurbitaceae, Lamiaceae and Oleaceae use raffinose family oligosaccharides in a transport mechanism known as polymer-trapping loading, which in a limited number of plant families is the main form of carbon transport [88] and renders such species highly efficient in water use. Subsequent to sugar transport, there are clear differences in sugar unloading strategies at sink organs that are paralleled with different relative activities of sucrolytic enzymes [89]. Moreover, a number of lineage-specific amino acid biosynthetic pathways have been identified, such as in the tomato wild species *Solanum pennellii*; here, unlike in the cultivated tomato, ISOPROPYLMALATE SYNTHASE 3 (IPMS3) is inactive, resulting in altered amino acid derived acyl sugars [90], while aromatic amino acid-derived compounds are also found in a more phylogenetically restricted manner such as betalin [91] or tyrosine and gallate conjugates [92], as well as in a dual route of lignin biosynthesis [93]. In all cases, these innovations appear largely to be driven by adaptive evolution. Of course, evolution of plant metabolism has not only involved gain- and change-of-function but also loss-of-function. Sticking with amino acid metabolism, the loss of phenylalanine hydroxylase is one such example [94]; another is the loss of the capacity to synthesize vitamin B_12_, an event that is considerably better understood [95]. Similarly, convergent evolution, which is often seen in specialized metabolism, has also recently been observed to be in action with regard to sphingolipid desaturation [22].

While the evolution of the above-mentioned pathways has been governed by natural selection, recent advances driven by the widespread adoption of metabolomics and next-generation sequencing have facilitated the study of a specific variant of evolution—namely artificial selection. In this vein, the processes of domestication and crop improvement, which are well-documented to result in genetic bottlenecks that massively reduce allelic diversity [96], can be widely addressed. This is a considerable advance relative to what was possible a few years ago, which was largely confined to the study of single metabolic traits that have profound effects on our foodstuffs, such as the century-long breeding pursuit of attractive colour and fragrance alongside reduced bitterness and altered sweetness, starchiness and acidity [97–102]. Although these studies were effective in identifying the genomic regions or even the genes responsible for defining these traits, the combination of metabolomics and next-generation sequencing has arguably allowed a greater advance in our understanding of how domestication and crop improvement have altered plant primary metabolism. Beleggia *et al*. [103] performed the first study at this level evaluating the metabolic changes that occurred during wheat domestication by investigating the relative levels of 51 core metabolites in kernels from three *Triticum turgidum* L. subspecies—namely wild emmer, emmer and durum wheat. This study revealed that the primary domestication—that of emmer—was marked by a decrease in unsaturated fatty acids, while a decrease in amino acid levels characterized the secondary domestication, that of durum wheat. Considerable alterations were also seen in metabolite correlation networks, suggesting that the domestication results in a deep restructuring of metabolism. Since this landmark publication, a number of other studies have been carried out at a large scale including those in rice, barley, tomato, watermelon and lettuce that were reviewed recently [7] alongside subsequent studies on coconut [104], jejubu [105] and peach [106]. Intriguingly, the changes that occur on domestication bear little relationship across species, even when comparing closely related ones, suggesting that they largely occur in a species-specific manner; it further suggests that considerable work will be required to understand the effects of domestication on the metabolism of each crop empirically.

## 2. Evolution of phytohormone biosynthesis, perception and signal transduction

Given that phytohormones play essential roles in normal growth and development they are often classified as primary metabolites, however, since many of them are synthesized from specialized metabolite precursors [107], we agree with Kliebenstein and Erb´s contention [108] that the line between primary and specialized metabolites is somewhat blurred. For this reason, we chose to describe the evolution of metabolite signalling separately. Thus, in this section, we describe the evolution of the classical phytohormones as well as those that were more recently discovered such as, for example strigolactones [109]. We additionally include discussion of other signalling pathways, here including calcium [110] and phosphate signalling [111] and that of plant growth regulators that are, as yet, not recognized as hormones in their own right [112]. We also here include discussion on ecological aspects of the evolution of metabolism, including those involved in mutualistic symbioses [113,114] and pathogenesis [115] as well as vegetative desiccation tolerance [116] and the influence of epigenetics on evolution [117,118].

Starting with the classical hormones, we here first turn to abscisic acid (ABA). ABA is critical in desiccation responses, and some ABA biosynthetic and signalling pathway genes are already present in streptophyte algae [47]. However, 9-*cis*-epoxycarotenoid dehydrogenase, the committed enzyme of ABA biosynthesis in land plants, is absent in charophytes. Yet, it is important to note that recently a novel route to ABA has been reported [119], which has not been investigated for its phylo-diverse distribution as of yet. Regarding its signalling, the ABA receptor might have been acquired through HGT from soil bacteria to an ancestor of Zygnematophyceae and land plants, with many—but not all—zygneamtophyceaen genomes coding for an orthologue of the PYR/PYL proteins [48,50,120,121]; while zygnematophyceaen PYR/PYLs are clearly orthologous to the ABA receptors of land plants, functional characterization has revealed that their regulatory effect on the Protein phosphatase 2C (PP2C; the next step in the ABA signalling cascade) is independent of ABA [122]. Regarding auxin biosynthesis, a similar situation to that of ABA is observed for the primary auxin indole-3-acetic acid [47,123]. The nuclear auxin response pathway hinges on a set of auxin response factors (ARFs) that have diversified into classes A and B, with class C ARFs likely being ancient [124–126]. By contrast, although the early steps of jasmonic acid (JA) biosynthesis leading to the key intermediate 12-oxophytodienoic acid (12-OPDA) are present in streptophyte algae [47], downstream enzymes are missing in green algae and even in the liverwort *Marchantia polymorpha* [127,128] and the moss *Physcomitrium patens* also appears not to produce JA, but is capable of producing 12-OPDA or *dinor*-OPDA (*dn*OPDA) and other oxylipins [129,130]. Stepwise recruitment of JA biosynthetic enzymes also appears to be involved in the stereo-specific production of JA [1], which is additionally characterized by ligand receptor co-evolution [131]. The JA signalling cascade is conserved in the liverwort and other land plant genomes, as well as functional in *M. polymorpha* [127,132]. In streptophyte algae, it appears not to be conserved [47], yet a CORONATINE INSENSITIVE 1 (COI1)-independent signalling pathway for temperature response has been identified in the alga *Klebsormidium nitens*, which appears conserved in land plants [133]. A comparative analysis of metabolic pathway genes across 72 genomes from green algae to angiosperms revealed that seed plants possess a complete set of genes encoding brassinosteroid biosynthesis and inactivation but ferns and lycophytes only harbour partial sets and bryophytes and green algae only have the initial pathway gene [134]. However, since brassinosteroids also occur in non-seed plants [135], this would appear to be by an unknown pathway with the known pathway evolving in a stepwise manner. The evolution of both gibberellic acid (GA; [136]) and ethylene [137,138] biosynthesis and signalling also followed such stepwise evolution. Salicylic acid (SA) has at least two main sources: isochorismate and benzoic acid [139–141]. Independent of the preferred source, all pathways are generally conserved across the green lineage, with the peculiar exception of the water fern *Azolla,* which is special because it lives in a permanent symbiosis with a nitrogen-fixing cyanobacterium and lacks any trace of isochorismate synthase, thus likely being unable to produce isochorismate itself [142]. SA perception and signalling are just now being analysed for their evolutionary distribution, and while NONEXPRESSER OF PR GENES (NPR) candidates exist in most land plant lineages (except the hornworts), functional testing of *M. polymorpha* NPR candidates rather suggests that they act as repressors similar to NPR3 and 4, rather than the positive regulator NPR1, suggesting that a signalling network diversified during the evolution of land plants [143,144].

Other non-phytohormone signalling pathways that have been studied in detail at the evolutionary scale are calcium and phosphate signalling, with work from the Abel group spanning both topics [145]. Calcium signalling is an ancient molecular mechanism that coordinates a wide range of developmental processes to environmental cues dating from before the dawn of land plants [146]. Intriguingly, a genetic screen for regulatory factors of glucosinolate homeostasis in Arabidopsis [147] identified a calmodulin binding protein with similarity to SF16 from sunflower [148], which they termed IQD1 owing to its respective arrangement of calmodulin recruitment motifs. A detailed analysis of the *IQD* gene families of Arabidopsis and rice included plant-specific domains with comparative phylogenetic analysis, suggesting that the major *IQD* gene lineages originated before the monocot–dicot divergence. The extant *IQD* loci in Arabidopsis resulted primarily from segmental duplication and preferential retention of paralogous genes, as is characteristic for regulatory proteins. In addition to a role in glucosinolate homeostasis, more recent work has pinpointed that genes of this family play a role in preprophase band formation and division-plane orientation [149], and it would seem highly likely that their role in glucosinolate homeostasis is a relatively recent innovation given the fact that these metabolites are not biosynthesized widely across the green lineage [147].

Similarly early-evolving was Fe-dependent phosphate signalling, with *LOW PHOSPHATE ROOT 1 (LPR1*) typifying an ancient Fe-oxidizing multi-copper protein family member that evolved early upon bacterial land colonization [111]. Indeed, as supported by phylogenomics, homology modelling and biochemistry, the ancestor of streptophyte algae and embryophytes likely acquired LP1-type ferroxidase from soil bacteria via HGT. On the basis of comparison with the metabolic lifestyle of extant sister genera, Naumann *et al*. [111] proposed that Arabidopsis LPR1 monitors subtle concentration differences of external Fe as a Pi-dependent cue to adjust root meristem maintenance. They also suggest that the acquisition of bacterial LPR1-type ferroxidase by embryophyte progenitors facilitated the evolution of local Pi sensing and acquisition during plant terrestrialization.

Twin papers in 2008 presented the structural elucidation of the novel phytohormone class of the strigolactones [150,151]. In the interim, considerable studies have been carried out with regard to these hormones and more phylogenetically restricted related compounds. These studies highlight the importance of strigolactone and the structurally related karrakin response pathways in various biological processes including the promotion of symbiotic interactions [114]. In a similar vein, the importance and emergence of metabolic immunity have been greatly advanced by modern methods of studying the evolution of metabolism [115], the evolution of vegetative desiccation tolerance [116] and the impact of epigenetics in phenotypic plasticity and adaptation [117,118]. Next, we will attempt to summarize the evolution and massive expansion of metabolism associated with plant specialized metabolism.

## 3. The radiant evolution of plant specialized metabolism

Having established a core metabolism, several factors have been identified that led to the vast metabolic diversity of plant specialized metabolism across various plant lineages. These include: (i) alterations in promoter strength resulting from differences in methylation or copy number within the promoter; (ii) single nucleotide polymorphisms in the coding region that effect enzyme activity, substrate specificity or both; (iii) polymorphisms resulting in a premature stop codon; (iv) gene fusions; (v) large gene deletions or insertions caused by transposon activity; or (vi) tandem gene duplication [152]. Comparisons across the domains of life have suggested that gene duplication is exceptionally prevalent in plants [153,154], which then acts as an initial step for the co-option or hijacking of (repurposed) core metabolic enzymes into specialized metabolism, leading to an unprecedented expansion of the plant chemical repertoire [155–157]. Here, we will define the major mechanisms by which this was achieved, paying particular attention to metabolic innovations that are believed to have been crucial for the thriving of plants in their terrestrial environment.

The importance of tandem duplication in the evolution of metabolism was demonstrated in an early study of the taxonomically restricted glucosinolate polymorphism in Arabidopsis encoded by the AOP2/3 and MAM1/3 tandem duplication interval [158]. More recently, a similar event was demonstrated to lead to the innovation of the saignols—a novel accession-specific set of phenylacetlyated flavonoids encoded within a serine carboxypeptidase-like tandem duplication [159]. Such events are presumed to be owing to a recent neofunctionalization event since they exhibit genetic polymorphism among natural accessions and are only conserved in the most closely related species [152]. Plant specialized metabolism is more permissive of mutations than its more evolutionarily conserved counterparts in primary metabolism [160]. This likely contributes to the massive chemodiversity of the plant kingdom. It is important to recognize that substrate specificity is only functionally relevant in the presence of alternative substrates at appropriate concentrations, i.e. the cellular context is extremely important in moulding the metabolic diversity of a species [161]. In this vein, it is important to remember that enzymes and metabolic diversity coevolved hand-in-hand in a stepwise manner with the tremendous expansion and diversification of enzyme families, for example P450 oxygenases, acyltransferases and *O*-methyltransferases, being facilitated by the availability of novel metabolites on which promiscuous enzymes acted and further specialized to use these new substrates [15]. We briefly outline the evolution of polyphenols, including the specific cases of lignin and red pigments, as well as alkaloids and terpenes in the following paragraphs.

It would seem natural to start with the phenylpropanoids. Not only are they by far the most studied class of specialized metabolites at the evolutionary level probably as a result of their importance in the terrestrial environment in conferring both UV tolerance and tolerance/resistance to biotic threats as well as the ability to stand vertically [159,162,163]. Massive gene duplication and neofunctionalization recurred across the diversity of enzyme families involved in the biosynthesis of phenylpropanoids [9,164,165]. This is true both for the establishment of major subclasses of flavonoids such as the isoflavones of legume and wheat [161,166] and in decorative reactions such as the production of the flavone wogonin in *Scutellaria baicalensis* [167] or in the convergent evolution of rosmarinic acid in Lamiacaea and Borginaceae [168]. Similarly, lignin—a class of 4-hydroxyphenylpropanoid-derived biopolymers—is uniquely and ubiquitously associated with vascular plants [169]. It provides structural rigidity and allows specialized cells to withstand negative pressure associated with long-distance water transport as well as providing a remarkable physico-chemical barrier offering protection from coevolving terrestrial animals and microbes. Its evolution is not only important for the domination of terrestrial ecosystems by plants. In addition, the huge amount of fixed carbon stored in lignin, coupled to a lag in the evolution of microbial enzymes that can efficiently degrade it, are major factors contributing to the considerable increases in the O_2_/CO_2_ ratio in the late Paleozoic era [170,171]. Lignin is largely composed of three p-hydroxyphenyl (H), guiacyl (G) and syringyl (S) units. H and G lignins are present in all vascular plants, while for S lignin there is high variation among and possibly even restriction to certain taxonomic groups, including flowering plants, certain lycophytes and some species of ferns and gymnosperms [163,172,173]. Lignin emerged from ancestral phenylpropanoid metabolism that became established in land plants approximately 500 Ma [163]; however, lignin-like phenylpropanoids have been reported in streptophyte algae [160], which suggests that it might have a much earlier evolutionary origin in a much deeper ancestor. The stepwise evolution of lignin has been the subject of recent reviews [163,169] and is also discussed by Kunz *et al.* [174] in this issue, so we will therefore not further detail it here. Suffice to mention that a recent study (published since these reviews) revealed that a *bona fide* HYROXYCINNAMOYL-CoA:SHIKIMATE HYDROXYCINNAMOYL TRANSFERASE that performs a critical function during seed development is functionally conserved in *P. patens* [175]. Moreover, metabolic analysis of loss-of-function mutants reveal that it is necessary both for the formation of caffeate derivatives and an intact cuticle [175]. Like lignin, the evolution of red pigmentation in land plants has been considerably studied. Production of phenylpropanoid-derived pigments is a common response to abiotic and biotic stress in the green lineage, but the exact type of pigmentation varies across plant lineages [176]. The majority of species are pigmented by flavonoids, including 3-hydroxyanthocyanins, sphagnorubins and auronidins, which are the predominant compounds conferring a red hue to flowering plants, ferns, mosses and liverworts, respectively [176,177]. However, some flowering plants have lost their ability to produce anthocyanins and rather produce betalins instead [178], whereas some terrestrial algal species produce red pigments that include carotenoids and phenolics [179]. Considerable study at the evolutionary level suggests that pigment biosynthetic pathways have evolved multiple times in land plants [176,179] and has started to provide potential explanations for the lack of red pigmentation seen in hornworts: All land plant groups—with the exception of the hornworts—produce flavonoids exhibiting ortho-hydroxylation of the B-ring which in all known cases is catalysed by FLAVONOID 3´HYDROXYLASE while some angiosperms additionally harbour the related enzyme FLAVONOID 3`5` HYDROXYLASE. Piatkowski *et al*. [180] identified that the orthogroup to which these enzymes belong is conserved across the green lineage, albeit several enzymes up- and/or downstream in the flavonoid biosynthesis pathway appear only to be present in seed plants. Contemporaneously, Zhang *et al.* [181] found that the CYP71 subfamily was greatly expanded in *Anthoceros angustus,* yet there is an absence of convincing FLAVONOID 3`(5`) HYDROXYLASE candidates in hornworts. However, our own analysis placed 72 Anthoceros CYP sequences within a larger clade [164], suggesting that bryophytes may have evolved alternative hydroxylation activities that operate upstream of chalcone synthase. This is, however, in no way a specific finding, with it being often remarked that flavonoid secondary modification varies both between and within the major land plant lineages [182,183].

Tandem duplication has also played important roles, with recent studies identifying that an atypical polyketide synthase coupled to P450-mediated cyclization led to the innovation of tropane alkaloids [184], while study of the chlorination of alkaloids in Menispermaceae uncovered an example of parallel cross-kingdom evolution [185]. Often co-clustering with CYP-enyzme-encoding genes are uridine diphosphate glycosyl transferase (UGT)-encoding genes [186]. These gene clusters have been observed both in *Medicago* and *Arabidopsis* [186,187]. They likely originate by tandem duplication forming several different clusters of lineage-specific size [188], which suggests a lineage-specific evolutionary history for them. While it is not yet clear whether these UGT clusters contribute to metabolomic diversity, it was recently suggested that exactly such redundancy (as also observed for other pathways, including routes to monolignol or epicatechin [189–192]) may allow for evolutionary flexibility while reducing the risk of losing essential metabolites [188].

Isoprenoids and terpenoids constitute a vast category of compounds. They encompass various essential molecules for the biology of plants and algae, such as carotenoids, phytols, retinols, tocopherols, dolichols, chlorophylls and squalene. Additionally, the phytohormones cytokinins and brassinosteroids are among the isoprenoid-derived compounds. As such, this group of compounds plays a pivotal role in regulating diverse aspects of growth, development and stress responses in embryophytes. Terpenoid biosynthesis involves the condensation of dimethylallyl pyrophosphate/isopentenyl pyrophosphate [193]. Notably, different organisms utilize distinct biosynthetic pathways: the mevalonate (MVA) and the methyl-erythritol 4-phosphate (MEP) pathways [194]. Archaea, certain eukaryotes and some Gram-positive bacteria follow the MVA pathway while, in contrast, Gram-negative bacteria, some Gram-positive bacteria and chlorophyte algae exclusively employ the MEP pathway [70]. Most astoundingly, embryophytes employ both the cytosolic MVA pathway and the plastidic MEP pathway inherited from cyanobacterial plastid progenitors. Some streptophyte algae have a complete MVA pathway, particularly Chlorokybophyceae, Klebsormidiophyceae and Zygnematophyceae, while all streptophyte algae exhibit partial MVA pathway presence; notably, the key enzyme 3-hydroxy-methyl-glutaryl-CoA reductase [195] is present in embryophytes and specific streptophyte algae. Thus, the co-occurrence of MVA and MEP pathways in streptophytes remains unclear (see also discussions in Rieseberg *et al*. [9]).

## 4. Methods facilitating recent understanding of the evolution of plant metabolism

While the above sections have described the evolution of plant primary, hormonal and secondary metabolism, respectively, these are quite diverse from one another. What are less diverse are the tools used to analyse them. As we stated in §1, these can be split into five groups: (i) the use of next-generation sequencing data; (ii) combining phylogenomics and synteny analyses; (iii) metabolomics; (iv) the approach of ancestral protein reconstruction; and (v) co-expression analyses. The first three approaches are widely used in the evaluation of the evolution of plant metabolism and have been relatively recently reviewed [196] but the final approach is arguably underused to date [18].

Given that Jacobowitz and Weng [196] provide an excellent review of the use of next-generation sequencing as a route to discover new plant chemotypes and as such uncover evolutionary aspects of pathways underlying the formation of lyciumin cyclic peptides [197], cyclotides and aromatic amino acid decarboxylases [198,199], as well as the metabolism-related analyses that can be achieved from the data assembled by the 1000 transcriptome project [20], we will not detail it here. Suffice to say that, as this special issue will stand testament to, next-generation sequence data are the key drivers to the tremendous leap in our ability to document the evolution of plant metabolism in recent years. Similarly, its use in phylogenomics and synteny studies is of vast importance here with computational developments being particularly important facilitators in this respect. Examples of note here are the use of phylogenomics to narrow down the number of candidate genes for a particular function, with the identification of salidroside biosynthesis in golden root [200], CYP71s as key oxidases in the biosynthesis of azadirachtin in *Azadirachta indica* [201] and styrylpyrone synthases involved in kavalactone biosynthesis in kava [202] and providing representative examples of the power of the phylogenomic approach. At a broader level, expanding the number of species that can be computationally analysed allowed a major study of the evolution of the polyketone synthase family, to which styrylpyrone synthases belong [21]. The combined phylogenetic and synteny analyses in this study provided insights into changes in the genomic location and context that are retained for a longer time-scale with more recent functional divergence captured by gene sequence alteration, suggesting that macroevolution of this superfamily was governed by whole genome duplications and triplications.

Metabolomics has also greatly advanced our understanding of metabolic evolution largely by means of metabolic analysis of natural variation, most recently including genome-wide association studies as a means to identify the genes associated with the abundances of a broad range of specialized metabolites [152,203]. These studies have also proven highly effective in identifying metabolic gene clusters in plants [204], which often prove to be an important starting point in understanding the evolution of the underlying pathways. That said, our level of coverage of the chemodiversity of plant metabolites—wich are estimated to number over 1 million [78]—remains relatively sparse [205] and despite the development of public spectral databases [206,207] and computational algorithms [208], this clearly limits the current scope of their use in evolutionary studies. However, one should not be too negative as novel approaches based on linking fragments of metabolites as a route to define metabolites of the same pathway appear to be very promising [209] and the numbers of metabolites that we can annotate is an order of magnitude higher than it was 20 years ago [205] . Similarly, while substantial recent effort has been placed on understanding the interactions of plant proteins with their molecular partners, relatively few studies in plants—by contrast to work in other organisms—address how these interactions evolve. An exception to this is a recent thought exercise in describing why some enzyme–enzyme interactions become obligatory, i.e. result in multi-enzyme complexes, while others such as metabolons remain transitory [210]. It is generally thought that ancestral proteins were more promiscuous than evolutionarily younger proteins and that specificity often evolved following gene duplication and subsequent functional refinement. However, ancestral protein resurrection studies have found that some of the evolutionarily younger proteins have evolved de novo from ancestral proteins lacking those functions. Intriguingly, such new interactions often evolved as a consequence of just a few mutations and, as such, acquisition of new functions appears to be neither difficult nor rare. However, only a few of them are incorporated into biological processes before they are lost to subsequent mutations [18]. At the same time, such interactions might arise through constructive neutral evolution [211]; the impact of such non-adaptative evolutionary processes also come to bear in biochemical pathways [212] and can provide the substrate for adaptive evolution [55]. In plants, a handful articles by the Barkman laboratory stand out. The first of these investigated the role of ancestral functional variation in determining modern-day protein specificity by looking at the SA/benzoic acid/theobromine lineage of enzymes. In doing so, they demonstrated that non-preferred activities were improved upon in daughter enzymes following gene duplication, suggesting a co-incidence with positive selection [24]. In a second study, they demonstrated that the convergent evolution of caffeine biosynthesis was the result of the co-option of exapted ancestral enzymes [25]. An earlier study that should be mentioned here is that of Des Marias and Rausher, who suggested that the escape from adaptive conflict hypothesis fitted the duplication of the anthocyanin pathway gene dihydroflavonol-4-reductase [213]. However, while intriguing, this hypothesis was questioned in a subsequent article by Barkman and Zhang, who posit that it is not clear that the acquired function was adaptive in either lineage [214]. Three further more recent studies merit discussion. First was the demonstration, via ancestral protein reconstruction, that chalcone isomerase arose from a non-catalytic ancestor, suggesting that enzymic innovation played a key evolutionary role in the expansion of plant flavonoid metabolism [215]. Second, the ancestral sequence reconstruction of the CYP711 family, which revealed that functional divergence in strigolactone biosynthesis—including both increased catalytic activity and stereoselectivity—was associated with gene duplication events in grasses [216]. Finally, an interesting classroom study published by Barkman and co-authors and analysing 76 enzymes of flowering plant species covering 23 orders and 41 families investigated how widely conserved substrate preference is for SA methyl transferase orthologues [217]. They found high conservation except following gene duplication and while these findings are exciting, as the authors themselves state, this is only the tip of the iceberg with thousands of sequences remaining uncharacterized in this enzyme family alone, suggesting that the power of this approach remains to be fully tapped.

Given the diversity and, in many cases, lineage-specificity of specialized metabolism, another area that is exploited is the co-expression of functionally related genes. Particularly, specialized metabolism genes that encode enzymes belonging to the same pathway appear (at least in parts) co-expressed [218]. Indeed, returning to lignin biosynthesis among several examples, Delli-Ponti *et al.* could show that the enzymes required in the process of lignin biosynthesis (at least one member of each enzyme family is required for the pathway to function) are all co-expressed and cluster within one module in a co-expression network [219]. The authors further highlight the potential of such analyses in identifying as-of-yet unknown genes from a given biosynthetic pathway in specialized metabolism. With our ability now to also perform such analyses in a comparative manner between closely and distantly related species [51,220], co-expression network analyses provide a tool to identify the conservation of: (i) biosynthetic pathways, (ii) identification of alternate lineage-specific deviations from otherwise conserved biosynthetic routes, and (iii) variation in regulatory patterns. As for many areas of biology, the power of machine learning as a means to enhance our understanding of the evolution of metabolism [221,222] is an area of high excitement and one we will return to in the concluding article of this special issue [19].

## 5. Conclusion and intent

As our above review of the state-of-the-art summarizes, while considerable research focus has been placed on understanding the evolution of energy metabolism in plants, with both the varying modes of photosynthesis and novel plant-specific aspects of respiration achieving considerable attention, that of other areas of plant metabolism is relatively poorly studied. With this collection of articles, we intend to focus on the evolution of the pathways of plant metabolism aside from primary energy metabolism. We will look at the intimately associated pathways of sugar, lignin and amino acid metabolism and the evolution of the major pathways of plant specialized metabolism that afford plants their exceptional chemical diversity. Where possible insights will be provided into the selective pressure driving the underlying evolutionary processes. The majority of articles will focus on specific pathways; however, the collection also includes some general methodological articles that are not chemically restricted and a handful of articles with a narrower taxonomic scope that cover major evolutionary changes in metabolism at a greater level of detail. Universal to the articles is the impact that the technologies of next-generation sequencing and—to a lesse- extent—metabolomics have played in enhancing our understanding of the evolution of plant metabolism. Taken together, our aim is that this collection of articles will: (i) provide an accurate and updated synthesis of our current understanding of the evolution of metabolism; (ii) introduce state-of-the art approaches by which to evaluate this process; (iii) define key priority questions for the development of the field in the future; and (iv) establish a road map by which these questions can be addressed. Each of the following chapters, while being standalone articles, should also contribute to answering at least a subset of these questions. In the concluding article, we will provide a synthesis of their answers alongside a critical assessment of how successful we have been in achieving our aims.

## Data Availability

This article has no additional data.
